# Task-relevant social cues affect whole-body approach-avoidance behavior

**DOI:** 10.1038/s41598-023-35033-7

**Published:** 2023-05-26

**Authors:** Robin Welsch, Heiko Hecht, John Stins

**Affiliations:** 1 Department of Computer Science, Aalto University, Espoo, Finland; 2 Department of Psychology, Johannes Gutenberg-Universität Mainz, Mainz, Germany; 3 Department of Human Movement Sciences, Faculty of Behavioural and Movement Sciences, Vrije Universiteit Amsterdam, Amsterdam Movement Sciences, Amsterdam, The Netherlands

**Keywords:** Psychology, Human behaviour

## Abstract

Positively evaluated stimuli facilitate approach and negatively evaluated stimuli prompt avoidance responses, as typically measured by reaction time differences when moving a joystick toward the own body or away from it. In this study, we explore whether a whole-body response (forward and backward leaning can serve as a better indicator of approach-avoidance behavior; AA). Thirty-two subjects were presented with pictures of males and females with angry or happy facial expressions. Subjects had to perform approach or avoidance responses by leaning forward or backward, either based on the facial expression of the stimulus or the gender of the stimulus. Leaning responses were sensitive to angry faces for explicit decision cues. Here, angry facial expressions facilitated backward leaning but not when responding to the gender of the stimulus. We compare this to the established manual measure of AA and discuss our results with regard to response coding.

## Introduction

The distinction between approach and avoidance (AA) in goal-oriented behavior can be applied to various contexts of human behavior. Positively evaluated stimuli, e.g., a person smiling, can elicit an approach motivation, whereas negatively evaluated stimuli, e.g., cursing, typically triggers avoidance motivation^1^. AA has often been investigated using arm movements, such as pushing or pulling. Here, we explore whether a full-body AA motor response can resolve certain methodological and theoretical ambiguities associated with arm movements and, thus, might be a more powerful tool.

Since Lewin’s^2,3^ formulation of field theory, psychologists have tried to capture behavior-related AA motivation. Solarz^4^ studied AA-related behavior using reaction time (RT). He presented subjects with words with either positive (e.g., “Happy”) or negative valence (e.g., “Stupid”). Subjects then reacted with a previously learned set of arm movements as fast as possible. For compatible trials, subjects responded with a push of a lever to words of negative valence, which constituted an avoidance reaction, and with a pull of a lever to positive words (approach reaction). In incompatible trials, the valence and reaction direction mapping was reversed (push—positive, pull—negative). In such a setup, compatible trials are typically facilitated and thus produce faster RTs than incompatible trials.

However, note that arm movements can be ambiguous regarding AA. For example, when we touch a hot stove, we will show an avoidance response by pulling our arms towards ourselves and away from the danger. Likewise, grabbing a ripe fruit from a tree involves an arm movement toward the object, not toward the actor. Markman and Brendl^5^ have demonstrated this ambiguity in tasks employing arm flexion and extension. Subjects pulled or pushed a lever in response to positively or negatively valenced words that were either positioned behind or in front of the subject’s name in a 2D-virtual environment. Not the arm movement per se, which constitutes an embodied perspective to the AA response, but the position of the subject’s name (which could change depending on the arm movement) determined the direction of AA effects. In some cases, even manikins, moving forward and backward, can symbolically represent the self^6,7^. Therefore, the anticipated movement concerning the represented self may be critical in formulating the AA effect, thus highlighting the crucial role of cognition in disambiguating the context.

Eder and Rothermund^8^ demonstrated the implications of this ambiguity in response coding as they could detect AA effects on a horizontal plane when subjects moved a joystick to the left (e.g., labeled as away) or right (labeled as towards) in response to a stimulus word. Thus, arm movements in a particular direction may not be ideal for studying the nature of approach-avoidance motivations as they can rely on arbitrary evaluative coding, the link between response valence and stimulus valence, and not on distance change per se.

Rinck and Becker^9^ developed the Approach-Avoidance Task (AAT). They added a zoom feature to the lever task of Solarz^4^, which increased the size of the stimulus picture on a computer monitor when the subject pulled a joystick and decreased the size when pushing the joystick; disambiguating approach-avoidance interpretations. First, spider-phobic individuals showed a more pronounced avoidance reaction towards spider pictures in comparison to healthy controls. Second, compatible with Chen and Bargh^10^, these effects still emerged when the response cue was stimulus-irrelevant, for instance, when reacting towards the format of the picture (landscape vs. portrait). And third, the size of the RT asymmetries between approach and avoidance was correlated with the walking speed when approaching a real spider, which indicates that the relative speed of the reaction is linked to distance behavior (for reviews see Laham et al.^11^, or Phaf et al.^12^). Nevertheless, the zoom-function, established in most research with manual AA responses, changes only the visual angle of the stimulus serving as a proxy of distance while the participant remains stationary.

Another solution in the field of AA research is the application of more ecologically valid ways of stimulus presentation^13–15^ and richer behavioral responses, such as articulation dynamics^16^ or whole-body movements^14,17–21^. The latter type of response is usually realized with movement registration techniques, allowing for the fine-grained spatiotemporal analysis of postural and locomotor behaviors. Most of these rely on movements with a clear forward or backward directional component coupled to a stationary stimulus. These tasks have two profound advantages. First, the distance to the stimulus automatically increases or decreases (plus the associated change in visual angle), thus making them highly suitable to study AA tendencies in a more ecologically valid setting. Second, whole-body movements may carry relatively strong sensorimotor associations related to approach (forward) and avoidance (backward)^22,23^.

Indeed, several studies in motor control and affect have found evidence for approach-avoidance tendencies at the whole-body level that mimic (to a certain extent) the rich literature on AA with manual responses such as joysticks and keypresses. Over the last two decades, two new movement paradigms have emerged, one (a) that uses quiet standing as a dependent measure and (b) another that uses step initiation in a particular direction.

When investigating quiet standing, the instruction is typically to stand still and, at the same time, pay attention to a stimulus. The researcher then tries to establish whether some minor postural adjustments become apparent in response to the stimulus’s content (usually the valence), despite the instruction to stand still. It is unclear whether approach-avoidance tendencies are so powerful that they can automatically ‘push’ the actor in a particular direction, except for the unlikely case of standing at the edge of a cliff, which reliably induces backward lean^24,25^. This might explain why AA tendencies are not reliably found in this field of research. It should be noted that freezing (overall reduction in postural sway) is, in fact, more common, especially with unpleasant items^26^, than forward/backward displacements of the body.

For quiet standing, several studies have found evidence of spontaneous backward lean in response to unpleasant stimuli^27,28^, but there is only little evidence of the opposite; that is, forward lean to pleasant stimuli^22^. Concerning the latter, several recent studies have emerged that ask the subject to make a single step in the forward or backward or sideways^29^ direction, usually in response to an emotional stimulus assumed to act as a motivational trigger^22,30^, or in response to another (non-valenced) aspect, such as the gender of a face^20^. This type of research is rapidly gaining in popularity, partly due to the broader availability and ease of use in recording human motion. Innovative as these measures may be, they capitalize on very subtle effects on posture or asymmetric body movement. We explore whether a different motor response, namely the initiation time of leaning the body forward (or backward), might have methodological advantages that could push the field of AA in new directions. Before we describe this novel motor response, we first describe the more commonly used whole-body responses in greater detail.

Step initiation studies fare better in the context of AA research. They allow for a more natural comparison with manual AA tasks since subjects can likewise make a forward (‘approach’) or backward (‘avoidance’) step. The researcher can generate various stimulus-reaction assignments, allowing for a comparison of ‘compatible’ and ‘incompatible’ blocks. Note that compared to manual AAT with a zoom function, changes in visual angle of the stimulus are accompanied by a change of the distance itself, thus resolving any ambiguity with regard to AA stemming from the position of the self. But there are at least two drawbacks to this paradigm. First, the calculation of RT is based on a close inspection of the movement output, typically a continuous time-series of forces and/or the center of pressure (COP). The biomechanical transition from not moving to moving is considered the movement onset and is then used (when time-locked to stimulus onset) to calculate RT. However, in the case of forward or backward stepping, the fine motor adjustments needed to organize and execute the step require, by necessity, a *sideways* body weight displacement. To take a step, the stepping leg must be lifted, and the body weight must be shifted to the stance leg. This means that observed RT-values may capture this lateral dimension of movement onset, not its fore/aft component, as would be desirable for AA research. A second drawback is that backward stepping can be considered unnatural or odd since there is no visual guidance of the step in contrast to forward stepping. This might explain why compatibility effects typically emerge with forward steps but not with backward steps^19^, thus pointing to an unexpected asymmetry in the facilitation of the AA response, which stands in contrast to avoidance responses in quiet standing.

In the quest for a movement pattern that avoids some of these problems, we investigated a novel motor output measure that is intermediate between quiet standing and step initiation, namely instructed postural leaning in a forward or backward direction. Postural leaning is established by a rotation around the ankle and/or hips, producing an anterior (COP moves towards the toes) or a posterior (COP moves towards the ankles) body weight displacement. We reasoned that we could reliably determine RTs based on the onset of leaning since no lateral weight shift or a step back is required. Moreover, by direct instruction to lean the body, the measure should produce more sizeable effects than the slight unintentional body shifts associated with quiet standing.

Note that Eder et al.^22^ have explored seated leaning movements in a virtual reality environment. There, they could show that leaning forward and backward movements are facilitated as a function of approach (forward) and avoidance (backward) motivation even if stimulus distance did not change, thus, lending support to our investigation of whole-body leaning movements, i.e., leaning while standing. Adding to their study, we want to explore how whole-body leaning movements are influenced by automatic and deliberate processing of AA-relevant stimuli.

## Aims and hypothesis

In this study, we set out to evaluate AA in deliberate leaning. We base our exploration on two criteria. First, the propensity to approach or avoid a given target can be triggered by evaluative processes, e.g., when deliberately approaching a liked person such as a friend, or it can be triggered reflexively, e.g., by pulling the hand away from a hot surface. Thus, AA responses can differ concerning their level of cognitive control.

Evaluative and non-evaluative AA processes have been studied separately^7,10,12,15,31–34^. In evaluative experimental tasks, often referred to as explicit AAT, researchers instruct subjects to process the stimulus (e.g., a happy face) and to select approach or avoidance based on a cue in the stimulus (e.g., pull in response to happy people). In contrast, non-evaluative tasks, often called implicit AAT, instruct participants to respond to effect-irrelevant features (e.g., approach female people), which typically results in overall faster response execution^9,13,35^. However, few attempts have been made to compare explicit and implicit AA responses for their efficacy in capturing automatic AA-processes, and the ones who did yield inconsistent results with regard to finding AA effects on automatic evaluations^7,13,35,36^. Therefore, this study aims to compare the whole-body AA task (AAT) in the efficacy of capturing automatic AA responses to the established manual AA task using a joystick. This is realized by employing a version of the AAT with AA-related decision cueing; subjects respond depending on the facial expression of the stimulus (approach—happy; avoid—angry), and an AA-unrelated instruction where subjects respond to the perceived gender of the face (approach—female; avoid—male) for each response modality (manual vs. whole-body).

In the latter case, the prediction, based on automatic stimulus evaluation, is that the task-irrelevant feature of the face, the facial expression, will differentially affect the response speed of AA responses. Given the assumed automaticity of the AA response and the strong sensorimotor grounding of AA with forward and backward locomotion^22^, a leaning-based whole-body AAT should produce compatibility effects, irrespective of the instruction. We thus hypothesize that AA effects are found when subjects perform forward/backward leaning instructed by an AA-irrelevant cue, that is, the perceived gender of a face (H1).

Second, AA effects in manual tasks are often pooled for compatible and incompatible trials and when analyzed more granularly often show a symmetric pattern^6,13,37–39^, (but also see Duijndam et al.^40^, Schneider et al.^41^ for asymmetry); the facilitation of approach compared to avoidance movements toward positive stimuli is as large as the facilitation of avoidance compared to approach movements concerning negative stimuli. However, this is not the case for the step-based AAT^19^. We will therefore investigate whether AA effects, the magnitude of approach bias for happy faces, and the magnitude of avoidance bias for angry faces are symmetric for both AAT versions. We hypothesize that whole-body AA effects are not symmetric, but AA effects are symmetric in the manual task (H2). We explore other indices of AA effects (approach happy—approach angry) and how they are affected by our experimental manipulations.

## Results

### Data analysis

Of 10,240 (32 × 320) movements, 855(8.35%) had to be discarded from the analysis for the following reasons: 214 movements were deemed too fast (RT < 250 ms); 89 movements were too slow (RT > 1500 ms); the 552 remaining movements were made in the wrong direction. To allow for flexible trial-based modeling, we analyzed the data using a Bayesian linear mixed model. This approach allows us to estimate parameter values of effect sizes and quantify the uncertainty regarding these estimates based on the information in our data and the priors applied. We used brms^42^, a wrapper for the STAN-sampler^43^, for R (Version 4.2.2) to model our data. We applied normally-distributed priors (*M* = 0, *SD* = 50) on all population-level effects, with Cholesky priors on the (residual) correlation (η = 2), a *t*-distributed prior (*df* = 3, *M* = 0, *SD* = 100) on the varying-level intercept, to allow for thicker tails. These priors are only weakly informative and mostly help in the regularization of the posterior distributions. We computed 4 Hamilton–Monte-Carlo chains with 20,000 iterations each and 20% warm-up samples. Trace plots of the Markov-chain Monte-Carlo permutations were inspected for divergent transitions. All Rubin–Gelman statistics^44^ were well below 1.1, which indicates that the chains converged. We used effect-coding on categorical variables (e.g., 1, − 1). For model selection, we use the Widely Applicable Information Criterion (WAIC). The WAIC is a criterion that provides a measure of the goodness of fit of the model and takes into account the complexity of the model.

**Figure 1:**
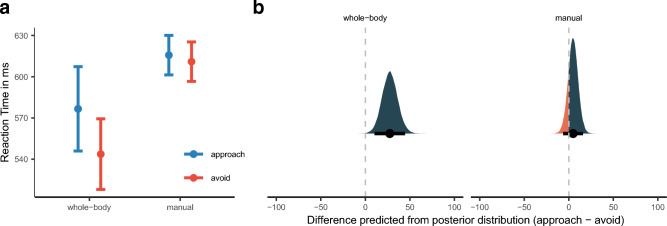
(**a**) Mean reaction time as a function of Reaction Direction as well as Response Modality. Error bars denote ± 1 standard error of the mean. (**b**) Posterior density plots with 95% high-density intervals, indicated by the error bars, and posterior medians for AA bias (approach minus avoid) for reaction time as predicted by the model for each Response Modality. The orange/blue area indicates the proportion of posterior samples opposite to the median and, thus, is a visual representation of the posterior median p-value. It quantifies the proportion of probability that the effect is zero or opposite given the data observed. The smaller the orange areas, the more reliable the estimation of the direction of the effect.

For statistical inference, following a Bayesian approach, we relied on (*p*_*b̃*_) and the high-density posterior intervals (HDI)*.* The posterior median *p*-value was computed by calculating the relative proportion of posterior samples being zero or opposite to the median (for a well-written and accessible introduction see Kruschke^45^). For an illustration of posterior distributions, see Fig. 1b. Thus, we quantified the proportion of probability that the effect is zero or the opposite of the medians sign, given the data observed. Note that this is the reverse of the frequentist approach to inferential statistics, where one measures the probability of the data given the Null-hypothesis concerning the test statistic. Still, *p*_*b̃*_ should have properties similar to the classical *p*-value^46,47^.

Effects were considered meaningful when there was a particularly low probability (*p*_*b̃*_ ≤ 2.50%) that the effect could be zero or the opposite. This threshold was chosen to resemble a conservative two-sided test with an alpha level of 5%, normally applied in classical statistical inference. In addition to the median of the parameter, we calculated the HDI at 95% of the posterior distribution for all parameters, which indicates the possible range of effects given the data.

Note that we have run a frequentist analysis before adopting the Bayesian approach. On a reduced set of participants and trials (2500 ms < RT < 250 ms RT), we have run repeated measures ANOVA models and *t*-tests on approach-avoidance bias scores. In summary, the analysis yielded a different pattern of results. It identified an avoidance bias for angry faces (Avoidance RT < Approach RT) that was based on main effect of Reaction Direction, i.e., a faster backward-leaning as compared to forward-leaning. This flawed but conventional analysis can be found in Supplementary Material 1.

Our Bayesian approach allowed us to quantify not only approach-avoidance bias scores but to inspect whether RT differs as a function of facial expression for each approach and avoidance movements for each decision cue and response modality. The Bayesian mixed model approach also allowed us to include the full dataset as it can handle missing data for participants more flexibly and allowed us to model whole-body and manual responses jointly. This increased the robustness of our model and also allowed us to better estimate the parameters in the model.

### Model building and selection

For our initial maximal model, we modeled the effect of all the experimental manipulations on RT as population-level effects (decision cue, response modality, facial expression and response direction), adding a varying intercept for every subject to account for the repeated-measures structure of the data in the mixed model. To allow for individual variation of effects in subjects, we added crossed-varying slopes of decision cue, response modality, facial expression and response direction. The varying intercepts and varying slopes for each subject serve the purpose of normalization and thus control for systematic individual differences on the dependent variable (e.g., individual overall variations in RT). All population-level effects of decision cue, response modality, facial expression and response direction were fully crossed in the model.

We compared this maximal model (WAIC = 120,771) to a model with the same population-level effects but a simpler varying-effects structure that only considered individual differences for facial expression and response direction that is more in line with the literature on AA effects. The simpler model (WAIC = 122,783) had a worse fit to the data as compared to the more complex model with the fully specified varying-effects structure. Further, we compared this to an even more simplified model resolving the term of response direction and facial expression to compatibility (compatible vs. incompatible trials; WAIC = 122,834). We also refitted the models with a studentized-link function, which allows for better handling of outliers. Here, the maximal model with a fully specified varying-effects structure with a studentized link-function (WAIC = 118,286) provided the best fit to the data. We, thus, analyzed only the posterior of this final model.

### Posterior of model parameters

Analyzing the population-level effects, we found a distinguishable effect for Decision Cue, $\widetilde{b}$ = 37.59 [29.69, 45.78]_,_ *p*_*b̃*_ = 0.00%*.* Explicit decision cues (*M* = 627 ms, *SD* = 106 ms) were processed slower than implicit decision cues (*M* = 548 ms, *SD* = 100 ms). We also found a distinguishable main effect of Reaction Direction, $\widetilde{b}$ = 8.07 [2.47, 13.48]_,_ *p*_*b̃*_ = 0.25% with avoidance movements being faster (*M* = 579 ms, *SD* = 97 ms) as compared to approach movements (*M* = 596 ms, *SD* = 105 ms), and an effect of Response Modality, $\widetilde{b}$ =  − 29.03 [− 50.10, − 8.08]_,_ *p*_*b̃*_ = 0.44%. Reaction times for the whole-body responses (*M* = 559 ms, *SD* = 157 ms) were, on average, faster as compared to the manual responses (*M* = 613 ms, *SD* = 81 ms). No main effects were found for Facial Expression, $\widetilde{b}$ =  − 0.54 [− 3.66, 2.58]_,_ *p*_*b̃*_ = 36.37%.

We found an interaction of Response direction and response modality, $\widetilde{b}$ = 5.65 [1.03, 10.39]_,_ *p*_*b̃*_ = 0.83%, see Fig. 1a. We followed this up with posterior predictive plots, calculating the difference between means for approach and avoidance movements for each Response Modality, see Fig. 1b. The mean difference (approach—avoid) for the whole-body task differed from zero, $\widetilde{M}$_pred_ = 27.50 [10.20, 44.60]_,_ *p*$_{\widetilde{pred}}$ = 0.01%, while the RT manual responses were symmetric for approach and avoidance responses, $\widetilde{M}$_pred_ = 4.78, [− 6.38, 16.00]_,_ *p*$_{\widetilde{pred}}$ = 19.03%; see Fig. 1b.

**Figure 2:**
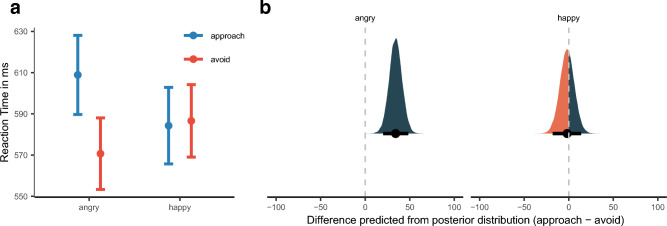
(**a**) Mean reaction time as a function of reaction direction and facial expression. Error bars denote ± 1 standard error of the mean. (**b**) Posterior density plots with 95% high-density intervals, indicated by the error bars, and posterior medians for AA bias (approach minus avoid) for reaction time as predicted by the model for each Facial Expression. The orange/blue area indicates the proportion of posterior samples opposite to the median and, thus, is a visual representation of the posterior median p-value. It quantifies the proportion of probability that the effect is zero or opposite given the data observed. The smaller the orange areas are, the more reliable the estimation of the direction of effect.

As expected, the interaction effect of Facial Expression and Response Direction surfaced (see Fig. 2a), $\widetilde{b}$ = 8.98 [3.80, 14.16]_,_ *p*_*b̃*_ = 0.05%, for posterior predictive plots on the difference in AA reaction times (approach—avoid) for each of the facial expressions, see Fig. 2b. The model predicted a mean difference for angry facial expression, $\widetilde{M}$_pred_ = 34.13, [20.12, 48.46]_,_ *p*$_{\widetilde{pred}}$ = 0.00%, but no difference for happy facial expression, $\widetilde{M}$_pred_ =  − 1.81, [− 18.03, 13.92]_,_ *p*$_{\widetilde{pred}}$ = 59.00%.

**Figure 3:**
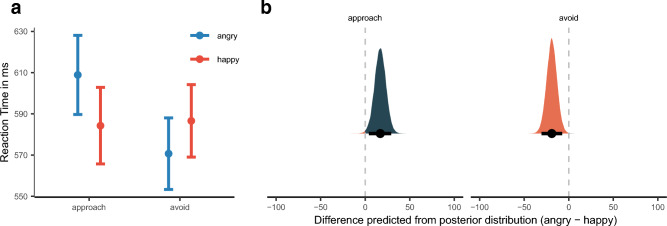
(**a**) Mean reaction time as a function of Facial Expression and Reaction Direction. Error bars denote ± 1 standard error of the mean. (**b**) Posterior density plots with 95% high-density intervals, indicated by the error bars, and posterior medians for facial expression bias (angry-happy) for reaction time as predicted by the model for each Reaction Direction.

The main effect of Response Direction and its interaction with Response Modality may confound the AA bias (see Fig. 3a) and, therefore, it is more appropriate to compare whether each Response Direction can differentiate for each Facial Expression, i.e., contrasting happy and angry facial expressions (angry—happy). Doing so, we find that the model can differentiate between Facial expressions for approach, $\widetilde{M}$_pred_ = 16.90, [4.05, 29.24]_,_ *p*$_{\widetilde{pred}}$ = 0.50% and avoidance reactions, $\widetilde{M}$_pred_ =  − 19.05, [− 30.60, − 7.40]_,_ *p*$_{\widetilde{pred}}$ = 0.01%, see again Fig. 3b. We will, thus, in the following only compare the effect of Facial Expression on each Response Direction as a means of quantifying the AA effect.

**Figure 4:**
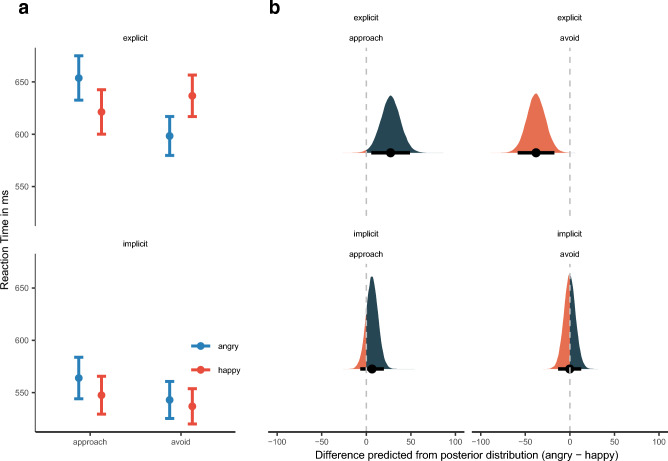
(**a**) Mean reaction time as a function of facial expression, decision cue and reaction direction. Error bars denote ± 1 standard error of the mean. (**b**) Posterior density plots with 95% high-density intervals, indicated by the error bars, and posterior medians for facial expression bias (angry minus happy) for reaction time as predicted by the model for each reaction direction and decision cue.

Response Cue, as a three-way interaction, further qualified the interaction of Facial Expression and Reaction Direction, $\widetilde{b}$ = 7.31 [1.95, 12.84]_,_ *p*_*b̃*_ = 0.48%. Descriptively, we can see in Fig. 4a that for explicit decision cues, the typical facilitation patterns surface: RT for happy faces with approach < avoidance and RT for angry faces with avoidance < approach. This is mirrored in the posterior predictive plot, Fig. 4b, we can see that only for explicit instructions avoidance movements, $\widetilde{M}$_pred_ =  − 37.97, [− 58.54, − 17.36]_,_ *p*$_{\widetilde{pred}}$ = 0.01% and approach movements can differentiate between facial expressions (angry–happy), $\widetilde{M}$_pred_ = 27.27, [5.53, − 6.67]_,_ *p*$_{\widetilde{pred}}$ = 0.09%, but not when the decision cue is implicit (approach; $\widetilde{M}$_pred_ = 6.46, [− 30.60, − 7.40]_,_ *p*$_{\widetilde{pred}}$ = 16.3%; avoidance: $\widetilde{M}$_pred_ =  − 0.20, [− 13.27, 12.71]_,_ *p*$_{\widetilde{pred}}$ = 48.80%.

We did not find a four-way interaction of response modality, response direction, facial expression and decision cue, $\widetilde{b}$ =  − 0.70 [− 6.03, 4.79]_,_ *p*_*b̃*_ = 39.83% (indicating that the data do not support H1). Response Modality was not a particularly informative factor for the discrepancy of explicit and implicit decision cues regarding the compatibility effect. Nevertheless, to directly test our hypotheses, we have computed mean differences for the factors of facial expression and response direction regarding decision cue separated for each response modality.

### Bias as a function of response modality and decision cue

**Figure 5:**
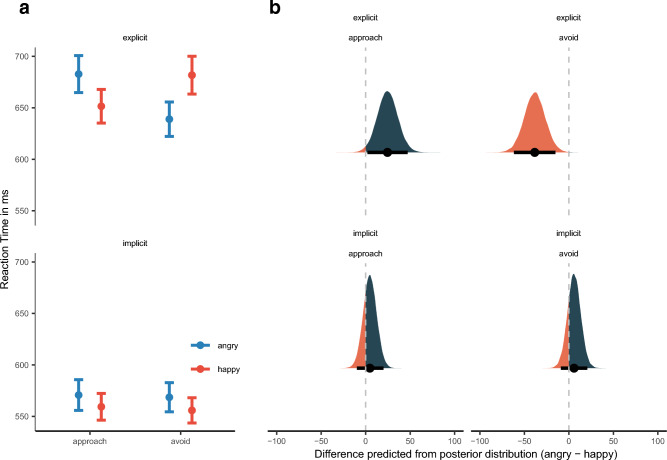
(**a**) Mean reaction time as a function of facial expression, decision cue and reaction direction for manual responses. Error bars denote ± 1 standard error of the mean. (**b**) Posterior density plots with 95% high-density intervals, indicated by the error bars, and posterior medians for facial expression bias (angry minus happy) for reaction time as predicted by the model for each Reaction Direction and Decision Cue for manual responses.

The manual response instructed by explicit cues could differentiate between Facial Expressions (Fig. 5b; approach: $\widetilde{M}$_pred_ = 24.52, [1.94, 47.31]_,_ *p*$_{\widetilde{pred}}$ = 1.73%; avoidance: $\widetilde{M}$_pred_ =  − 38.42, [− 61.72, − 15.19]_,_ *p*$_{\widetilde{pred}}$ = 0.08%) whereas implicit instructions could not (approach: $\widetilde{M}$_pred_ = 4.75, [− 9.81, 20.0]_,_ *p*$_{\widetilde{pred}}$ = 26.15%; avoidance: $\widetilde{M}$_pred_ = 5.80, [− 9.04, 20.67]_,_ *p*$_{\widetilde{pred}}$ = 21.54%). Reaction times were all around 570 ms for the implicit manual AAT, see Fig. 5a.

**Figure 6:**
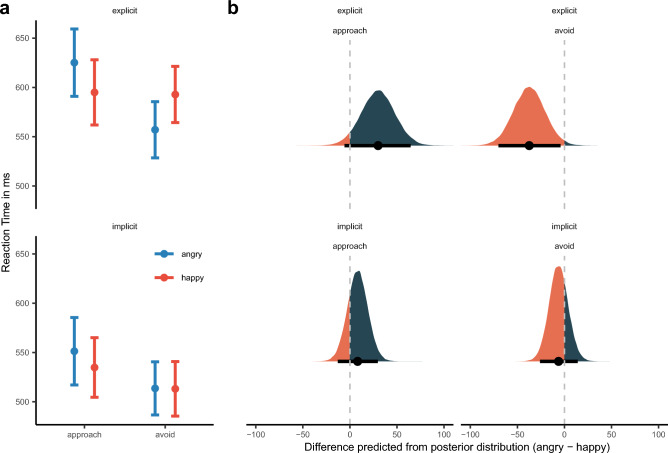
(**a**) Mean reaction time as a function of facial expression, decision cue and reaction direction for whole-body responses. Error bars denote ± 1 standard error of the mean. (**b**) Posterior density plots with 95% high-density intervals, indicated by the error bars, and posterior medians for facial expression bias (angry minus happy) for reaction time as predicted by the model for each reaction direction and decision cue for whole-body responses.

We find a different pattern for the whole-body AATs. When instructed by an explicit evaluative cue, the whole-body avoidance response can differentiate between happy and angry facial expressions, $\widetilde{M}$_pred_ =  − 37.41 [− 70.15, − 4.13]_,_ *p*$_{\widetilde{M}}$ = 1.34%, but this is not the case for approach responses, $\widetilde{M}$_pred_ = 29.98 [− 5.75, 64.58]_,_ *p*$_{\widetilde{M}}$ = 4.82%. The implicit whole-body AAT could not differentiate between facial expressions, (approach: $\widetilde{M}$_pred_ = 8.21 [− 12.92, 29.68]_,_ *p*$_{\widetilde{pred}}$ = 22.22%; avoidance: $\widetilde{M}$_pred_ =  − 6.25, [− 25.99, 14.13]_,_ *p*$_{\widetilde{pred}}$ = 26.83%). The data is, therefore, not in line with H1. Implicit instructions did not produce any AA effects for the whole-body AAT (Fig. 6).

To directly test H2, we computed the AA bias (approach angry—avoid angry; approach happy—avoid happy) for each facial expression and response modality and then multiplied bias scores for angry faces (approach angry—avoid angry) with − 1, so that bias is directed in the same direction; then we computed the difference of these bias scores from the posterior predictive distribution. If the AA response is symmetric, this difference in AA bias scores should be centered around zero. We found that for the explicit manual AAT and the implicit manual AAT, this comparison was centered at zero (explicit: $\widetilde{M}$_pred_ = 9.20 [− 20.62, 40.02]_,_ *p*$_{\widetilde{pred}}$ = 26.75%; implicit: $\widetilde{M}$_pred_ = 9.88 [− 15.59, 34.84]_,_ *p*$_{\widetilde{pred}}$ = 21.69%), indicating symmetry, while for the whole-body AAT, we found large differences (explicit: $\widetilde{M}$_pred_ = 44.52 [4.02, 86.24]_,_ *p*$_{\widetilde{pred}}$ = 1.50%; avoidance: $\widetilde{M}$_pred_ = 65.19 [22.63, 107.82]_,_ *p*$_{\widetilde{pred}}$ = 0.13%) indicating asymmetry, lending support to H2.

## Discussion

The newly developed leaning-based whole-body AAT was able to capture AA motivation in response to facial expressions for explicit evaluative decision cues but not for implicit instructions, that is, when subjects deliberately responded to the facial expression but not when responding to the gender of the face. This puts previous results obtained with the manual AAT into perspective. The latter only captured AA effects for the explicit condition, replicating our prior research^13^. The explicit manual AAT had the desirable property of symmetry, while the explicit whole-body AAT only differentiated happy and angry facial expressions for the avoidance response but not for the approach response, with a general faster avoidance—backward leaning—response. Therefore, compared to whole-body AATs based on stepping^17,19–21^ (for an exception see Eder et al.^22^), which could not find facilitation of avoidance (i.e., faster backward stepping to unpleasant items), the whole-body AAT based on leaning appears suitable to capture avoidance facilitation for angry facial expressions. The asymmetry of the AA response for the whole-body AAT can be explained by the differences in mobility when leaning backward and forward. Furthermore, we found no support for the assertion that AA effects in the whole-body AAT are stronger^22^ and could thus allow to capture AA effects for implicit decision cues. On average, the effect sizes were smaller than the effects of the manual AAT. The theoretical advantage in terms of sensorimotor grounding of AA with forward and backward locomotion^22^ did not surface for our implicit whole-body AAT when compared to a manual AAT.

Why was the response facilitation in the implicit AATs not replicated? Be reminded that in the explicit AAT, subjects had to react to the facial expression, whereas in the implicit AAT, subjects had to react to the gender of the avatar. Embodied accounts to approach-avoidance effects posit that arm flexion is facilitated in response to positive stimuli, and arm extension is facilitated in response to negative stimuli^10,11,48,49^, irrespective of the level of stimulus processing. However, the validity of these effects has been challenged by meta-analyses^11,12^ and direct replication attempts^31,50^. Our data align with this critique as we could not find AA biases for implicit decision cues, irrespective of Response Modality; that is we found no bias in leaning for implicit decision cues.

Notably, manual responses and whole-body responses markedly differ concerning what happens before the AA response. Whereas in the manual AAT, the hand is fixed to the joystick by the subject’s grip, in the whole-body AAT, subjects are standing and thus exert continuous control to maintain postural equilibrium. Quiet standing is accompanied by minuscule amounts of sway, mainly in the anterior–posterior directions. This sway being implicitly affected by AA motivation^24–26,28^ is well documented and was hypothesized to be a precursor for voluntary responses to facial expression. However, our Bayesian analysis shows that effects on voluntary leaning are non-existent or very small for implicit instructions. Thus, future research should investigate whether differences in spontaneous sway translate to effects on voluntary locomotion such as leaning and stepping.

On a cautionary note, most studies test bias in AA by calculating scores based on the difference of approach and avoidance for a set of stimuli. While this aligns with AA theory^2,3,51^ and the common practice of reporting manual responses^52^, we could illustrate in our Bayesian analysis how this can result in misleading conclusions when main effects of movement direction are present in a whole-body task, e.g., faster avoidance than approach responses. While our data shows that manual responses are not affected by this and produce symmetric AA effects, whole-body tasks need a more nuanced analysis to consider this potential confound in the scoring procedure.

One may have to take some limitations into account. For the whole-body AAT, the leaning movement was recorded as the change of COP from equilibrium to the anterior or posterior direction. This involves a displacement only along the main axis of the foot of the subjects. Therefore, other metrics frequently used to validate response times in stepping-based AATs^14^, such as movement speed and absolute levels of COP shift, were not analyzed. Note, however, that the manual AAT suffers from the same limitations by design, i.e., only response times can be captured. Future studies could employ body-tracking or head-tracking to capture voluntary leaning movements of the upper body, potentially enlarging the size of effects and allowing for the integration of metrics of speed and distance traversed. This could be realized in virtual environments where stimuli can be presented in 3D space. Welsch et al.^13,14^ provide a first implementation of a manual AAT and whole-body AAT that employed a stepping response. Therefore, the logical next step is investigating a whole-body AAT within an immersive virtual environment. This study is the first to show that rendered 2D-images of virtual characters can have a powerful effect (about 37 ms facilitation) on postural control. Until now, studies have used more naturalistic stimuli, such as photographs of faces^52–54^. The effect sizes of the AA response obtained with the avatars are comparable to studies based on photographic stimuli^11,54–56^, or based on verbal stimuli^10^ or symbolic representations^7^.

We have only used a very limited set of facial expressions (happy vs. angry) for two reasons. First, adding other facial expressions (e. g., disgust, sadness) to the design would have lengthened the experiment to become unwieldy. Second, when contrasting happy and angry facial expressions with other expressions, such as sad or fearful expressions, which share similar evaluative connotations, the latter can trigger simultaneous approach and avoidance-related behavior, depending on the contrast^34^ or context^57^. Thus, predictions in terms of symmetry and direction of effect would no longer be straightforward. Nonetheless, future studies that examine whole-body responses from an applied perspective should consider a large range of facial expressions. Including neutral stimuli will also allow for a very straightforward identification of general biases, such as faster backward leaning. Note also that our sample with a majority of participants identifying as female, could have exaggerated our effects, as a recent study finds better facial expression recognition in female participants^58^.

In sum, our findings show that facial expressions influence the control of deliberate leaning in a whole-body AAT. Backward leaning was strongly facilitated when viewing an angry facial expression that had to be evaluated (explicit decision cue). AA facilitation of leaning was only present when avoidance movements were based on facial expression but not when responding to the gender of the face. Thus, the whole-body AAT measure based on leaning, as pioneered with avatars in the present experiment, is a promising tool for studying avoidance facilitation in response to social threat. The combination of leaning and stepping movements allows us to capture both approach and avoidance facilitation in a whole-body AAT.

## Methods

### Subjects

Thirty-two individuals (24 identified as females; 8 identified as males; 0 non-binary; 0 other; 0 unspecified; *M*_age_ = 21.94, *SD*_*age*_ = 3.65) participated in the experiments. None of the subjects had neurological conditions that prevented them from performing the tasks. The local ethics committee (VCWE, Vrije Universiteit Amsterdam) approved the experimental protocol beforehand. All procedures performed with human subjects were in accordance with relevant guidelines and regulations. Informed consent following the declaration of Helsinki was obtained from all subjects included in the study. A sample size of *N* = 32 was approximated by a-prior power simulation^59^ of a three-way ANOVA model (*r* = 0.50; *SD* = 30 with *N* = 32), assuming a difference in response times by 50 ms in compatible trial pairings, happy-approach and angry-avoidance, as compared to incompatible trial pairings, angry-approach and happy-avoid, which yields a minimum power for the three-way interaction of 80%. The analysis for this model can be found in Supplementary Material 1.

### Whole-body AAT

We used a custom-made 1 m × 1 m strain-gauge force plate for the whole-body task to record the center-of-pressure trajectories. The sampling frequency was 1 kHz. The plate consisted of eight force sensors, four measuring forces in the z-direction (one in each corner), and two sensors each for the x and y directions (embedded in the four sides of the plate). Before the experiment, we calibrated the plate with a set of weights. The raw force traces of the eight channels, the summed forces in the three directions, and the COP time series (in the anterior–posterior direction and medial–lateral direction) were stored for further analysis. In the plate’s geometric center, we had attached small pieces of white tape, which marked the starting position from which each lean should be performed. A 55-inch monitor (Philips) was positioned 180 cm in front of the subject at eye level and was used to display the stimuli (38.3 cm height; 24.01 cm width; 12° visual angle vertically). As done in earlier experiments^17^, we used a small photodiode attached to the monitor to synchronize stimulus events with the force plate recordings. The data from this light sensor and the force plate were fed into an Anolog to Digital converter, which then sent the data to the measurement computer.

Using Psychtoolbox 3^60^ in Matlab 2014, we programmed brief light pulses coinciding with the main stimulus events, namely the fixation cross’s onset and the visual stimulus’s beginning. These light pulses allowed us to mark these events in the force plate data such that reaction times (i.e., the time difference between the visual stimulus and movement onset) could be calculated. The light pulse data were stored as a separate data channel; they have an exact square wave shape, signifying the onset and offset of each light pulse. We used a custom-made program to read the data, do the preprocessing, and extract reaction times. Using the light pulses, we isolated each leaning trial in the continuous data signal, allowing us to calculate reaction time.

**Figure 7:**
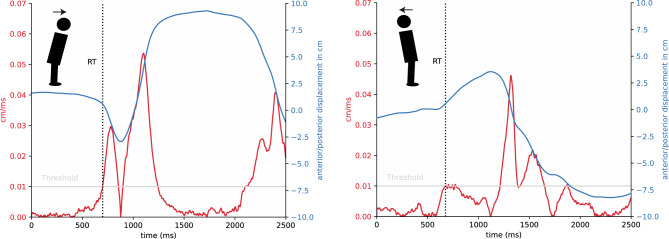
Exemplary trials for a participant encountering an angry face. Left: Trial for one subject leaning forward. Right: Trial of one subject leaning backward. Anterior–posterior displacement of the COP in cm (pos in blue) as a function of time for 2500 ms. Speed in cm/ms (red) as a function of time. The Grey horizontal line depicts the threshold of 0.01 cm/ms for reaction initiation. The black dotted line labeled “RT” indicates when this threshold has been reached (reaction time). Note that a postural lean in a particular direction is usually preceded by a small postural displacement in the opposite direction. This bi-phasic pattern emerges for biomechanical reasons and is related to the generation of momentum.

We calculated reaction time as the change from quiet standing (i.e., awaiting the stimulus) to movement onset. In some of our earlier papers with stepping^17,19^, we used a force threshold in the anterior–posterior direction based on the observation that forward stepping is characterized by an evident change in the force profile. However, in the current experiment, subjects merely had to lean, that is to tilt their body forward or backward, with minimal force changes. To this end, we decided to calculate RT based on the COP trace, more precisely, its change in velocity in the anterior–posterior direction. We defined reaction time as the time interval between stimulus onset and the moment when a speed of 0.01 cm/ms was exceeded, as illustrated in Fig. 7. Before this analysis, we applied a low-pass Butterworth filter (4th order, w = 0.09) and a rolling mean normalization on a window of 50 ms to the COP trace.

We used a custom-made Python (version 3.9.13) program to identify this time point based on the consideration that a change in COP dynamics from quiet standing to weight displacement during leaning is always accompanied by a substantial increase in the speed of the COP. Subjects had to stand still before the presentation of the image, with the arms relaxed alongside the body, and to initiate a lean forward or backward as soon as the stimulus appeared on the screen. No instructions on the lean extent and speed were given, but subjects had to keep the soles of their feet in contact with the plate. All leaning movements had to be initiated as fast as possible, and subjects then resumed their original position at their own pace to await a new trial. Participants completed four training trials before each task.

### Manual AAT

The AAT ran under Inquisit 4 (2014) and was completed on a 19-inch computer screen in a quiet room. Stimuli (initial size: 18.8 cm, height 13.29 cm width) were depicted on a Dell computer display (P190st) at eye-level; subjects sat in a chair at around 90 cm from the screen (12° visual angle vertically). For the Joystick task, a joystick (Thrustmaster T16000M) was mounted on a desk in front of the subject at a height of about 90 cm. With 16-bit-precision and a dead zone of 1% (the range of joystick positions not signaling movement) of the maximum joystick displacement, it was sufficiently accurate for our experiment.

In every trial, the task was to deflect the joystick in response to the stimulus. A pull or push of the joystick by one degree produced a dynamic zoom-in for the visual scene (i.e., back-and forth movement would have increased and then decreased picture size in the same trial) by either minimizing the picture by half or doubling the picture size, thus being presented in full-screen. We analyzed the RT from the picture’s appearance until 3° joystick displacement, with a precision of 1 ms. Participants completed four training trials before each task.

### Stimuli and design

**Figure 8:**
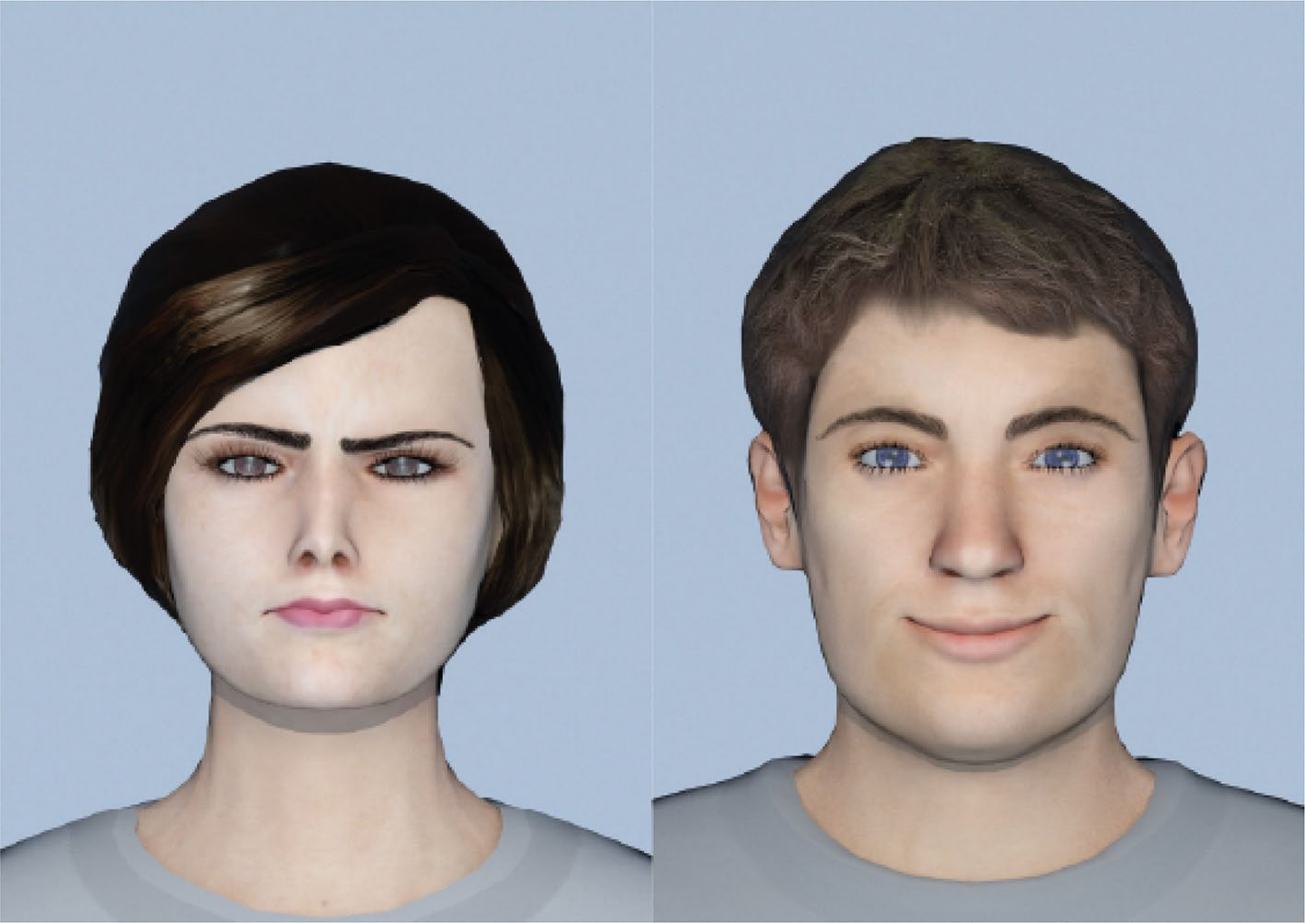
Two of the eight stimuli. Left: Female virtual face with angry facial expression. Right: Male virtual face with a happy facial expression.

Facial stimuli were taken from virtual people designed in Makehuman 1.1.0 Nightly Build 1.1.0^61^, and facial expression was modulated in 3DSMAX 2014^62^ to resemble Ekman pictures^63^. Welsch et al.^64^ let a different sample of participants rate these virtual people with each facial expression on the Self-Assessment Manikin-scales^65^ and could show large differences in valence (*d*_*z*_ = 1.66). Four different (two female, two male) Caucasian virtual persons were used to present a variety of social stimuli. The virtual faces had been previously used in similar AA-related studies and have reliably produced AA-related behavior^13,64,66^. Each of the four virtual faces was presented with both happy and angry facial expressions. We rendered an image of the virtual face from a frontal viewpoint, see Fig. 8. We used virtual faces as the mapping of facial expressions could be applied similarly to each character, thus ensuring that the expression was most similar across faces.

Subjects each performed 160 forward/backward leaning movements on the plate and 160 deflection movements with the joystick upon presentation of a visual stimulus. Following the convention in the AA literature, we label forward-leaning and pull of the joystick to a happy face as compatible. Likewise, backward leaning and push movements of the joystick in response to angry faces were also labeled as compatible. Conversely, forward-leaning and pulling the joystick in response to an angry face and backward leaning and pulling the joystick as a response to happy faces are incompatible. We presented two female and two male virtual people, each with a happy and an angry facial expression. Every stimulus was to be pushed and pulled five times in response to either the gender or the facial expression of the depicted face, depending on the instruction.

In the compatible AAT block, subjects avoided the angry-faced virtual person and approached the happy-faced virtual person. In the incompatible block, this mapping was reversed. In the AA-implicit condition, subjects had to react to the gender of the virtual person, thus, they did not have to attend to the facial expression shown in the picture. In one block, subjects performed approach movements in response to males and avoidance movements in response to females. In the other block, subjects performed approach movements in response to female faces and avoidance movements in response to male faces. Overall, subjects completed 320 trials each. These consisted of 4 Faces (2 male, 2 female) × 2 Facial Expression × 2 Decision Cue (gender vs. face of a virtual person) × 2 Response Modality (whole-body vs. manual response) × 2 Compatibility (e. g., pull/lean forward = happy vs. push/lean back = happy) × 5 repetitions. Trials were nested in counterbalanced blocks of response modality, decision cue, and compatibility. This means that subjects either completed all manual responses first and then completed all leaning responses on the forceplate or vice versa. Within these blocks, subjects either started with gender or facial expression as a decision cue, again counterbalanced and completed both compatible and incompatible blocks.

### Procedure

Subjects were told which lean, or movement (i.e., forward or backward) had to be performed in response to each of the faces (e.g., forward in response to male faces). The timing of stimulus events was as follows: each trial started with a fixation cross in the middle of the screen, with a duration of 2 s, then the imperative stimulus (the face) was shown for a fixed duration of 6 s for the whole-body response task and until a reaction occurred for the joystick response.

### Consent to participate

Informed consent was obtained from all individual participants included in the study.

## Supplementary Information

Supplementary Information 1.

Supplementary Information 2.

## Data Availability

All data generated or analyzed during this study are included in this published article and its Supplementary Information files.
