# Supplementary material for: Task-relevant social cues affect whole-body approach-avoidance behavior
Source: Sci Rep. 2023 May 26;13:8568. doi: 10.1038/s41598-023-35033-7 (PMC10219979; doi:10.1038/s41598-023-35033-7)
Supplement: Supplementary file 1 — Supplementary Information 1. [file 41598_2023_35033_MOESM1_ESM.docx]

# Results of alternative frequentist analysis

**[NOTE THAT THIS ANALYSIS IS FAULTY; WE ADDRESS THIS IN THE MAIN MANUSCRIPT]**

## Data analysis

Our initial screening of the data revealed that the data of 3 subjects could not be used due to a large number of incorrect leaning movements, highly atypical COP traces (perhaps suggestive of sub-movements and hesitation), or overall prolonged movement onset (RT). We decided to exclude these data from further analyses.

Of the remaining 9280 (29 ✕ 320) movements, a further 448 (4.83%) had to be discarded from the analysis for the following reasons: 184 movements were too fast (RT < 250 ms); 20 movements were too slow (RT >2500 ms ); 244 remaining movements were made in the wrong direction. We performed two mixed-factors analyses of variance (ANOVA) for each Response Modality (manual responses vs. whole-body responses) with the following factors: Response Direction (approach vs. avoidance movements; within-factor), Facial Expression (happy vs. angry faces, within factor), and Decision Cue (explicit vs. implicit decision cue, within factor).

To have an individual estimate of the effects, we calculated bias scores for each response modality and facial expression. We subtracted the mean of trials approach movements from the mean of the trials for every subject’s avoidance movement under each condition. Thus, the scores depict a bias score. Positive scores indicate a tendency to rather avoid than to approach the stimulus. Negative scores indicate a tendency rather to approach than avoid the given stimulus. The analyses were carried out in R ^1^ and test statistics were considered meaningful when crossing an alpha-threshold of 5%.

## Whole-body AAT

We found a significant effect of Decision Cue, *F*(1, 28) = 15.13, *p* < .001, *η*²_p_= .35, with the explicit decision cues produced faster RT (*M* = 521 ms, *SD* = 194) as compared to implicit decision cues (*M* = 583, *SD* = 165). There was also a main effect of Reaction Direction, *F*(1, 28) = 12.26, *p* = .002, *η*²_p_= .30. Leaning backward (avoidance, *M* = 530 ms, *SD* = 158 ms) produced relatively faster responses than leaning forward (approach, *M* = 575 ms, *SD* = 197 ms). A significant Reaction Direction ✕ Facial Expression interaction was found, *F*(1, 28) = 5.83, *p* = .023, *η*²_p_= .17. Compatible trials (*M* = 539, *SD* = 171 ms) were faster than incompatible trials (*M* = 565 ms, *SD* = 183). We found a non-significant three-way interaction, *F*(1, 28) = 0.98, *p* = . 332, *η*²_p_= .03*,* see also Figure 1 on top.

We tested bias scores against zero with a one-sample *t*-test, see Figure 2. A bias score deviating from zero indicates a significant facilitation in a given Response Modality and Decision Cue. For angry faces, we found a facilitation for explicit decision cues, *t*(28) = 3.23, *p* = .003, *d* = 0.60, but not for happy faces, *t*(28) = -0.54, *p* = .592, *d* = -0.10. The same pattern occurred for implicit decision cues. There was no bias toward happy faces, *t*(28) = -1.81, *p* = .081, *d* = -0.34 but a bias for angry faces, *t*(28) = 2.63, *p* = .014, *d* = 0.49.

## Manual AAT

The main effect of Decision Cue was significant, *F*(1, 28) 159.80, *p* < .001, *η*²_p_= .85. This effect was due to faster reactions when responding to the gender of the virtual person (*M* = 564 ms, *SD* = 77 ms ) as compared to the facial expression (*M* = 666 ms, *SD* = 94 ms). Most importantly, the compatibility effect emerged, as indicated by a Reaction Direction ✕ Facial expression interaction, *F*(1, 28) = 12.87, *p* < .001, *η*²_p_= .31. Compatible trials (*M* = 604 ms, *SD* = 84 ms ) were on average faster than incompatible trials (*M* = 625 ms, *SD* = 85 ms). In line with prior studies, the effect was qualified by a three-way interaction of Facial Expression ✕ Reaction Direction ✕ Decision Cue, *F*(1, 28) = 23.05, *p* < .001, η²_p_= .45, see the bottom of Figure 1.

Inspecting the bias scores (Figure 2), we find that both happy facial expression facilitates approach movements, *t*(28) = 2.52, *p* = .018, *d* = 0.47, and angry facial expression, t(28) = 5.09, *p* < .001, *d* = 0.94 facilitate avoidance respectively for the evaluative condition. However, none of the bias scores for happy, *t*(28) = -0.62, *p* = .540, *d* = -0.12, nor for angry, *t*(28) = -0.22, *p* = .828, *d* = -0.04, the facial expression is significant from 0 for implicit decision cues.

## Comparison of compatibility effects across Facial Expression

Comparing the RT bias (approach-avoid) for happy and angry faces for each Decision Cue and response modality, it becomes apparent that although the manual response for explicit decision cues may not have the desirable property of symmetry for angry and happy Facial expressions, this is not the case for the leaning-based AAT versions.

To quantify this, we multiplicated all scores for angry-faces with *-1 and ran a paired-sample *t*-test. There was no significant difference for happy and angry Facial expression biases in the explicit, *t*(28) = -1.37, *p* = .180, d = -0.26 , and implicit manual AAT, *t*(28) = -0.35, *p* = .725, *d* = -0.07, but a difference for the evaluative, *t*(28) = -3.16, *p* = .004, *d* = -0.59, and implicit leaning-based AAT, *t*(28) = -2.55, p = .017, d = -0.47**. [NOTE THAT THIS INTERPRETATION IS FAULTY; WE ADDRESS THIS IN THE MAIN MANUSCRIPT]**

**

explicit

*Figure 1. Raincloud plots for reaction time as a function of Facial Expression, Decision Cue (implicit: gender of the face; explicit; facial expression), Reaction Direction as well as Response Modality. Raw data are summarized in density distributions with box plots and raw data points underneath.*

Whole-body manual

Whole-body manual

explicit


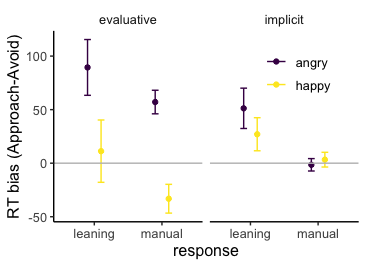
*Figure 2. Mean bias scores as a function of Facial Expression, Decision Cue and Response Modality. The grey horizontal line depicts a threshold of zero.*

1 R: A language and environment for statistical computing (R Foundation for Statisitcal Computing, Vienna, Austria, 2010).
